# Toward Sustainable Workplaces: The Roles of Work–Family Conflict, Social Support, and Meaning in Employee Well-Being and Performance After Parental Leave

**DOI:** 10.3390/bs16091566

**Published:** 2026-09-03

**Authors:** Cătălina Radu, Loredana Simona Berta (Carp), Adina-Raluca Olteanu, Miruna-Georgiana Stan, Eugen-Mihai Dragomir

**Affiliations:** Doctoral School of Management, Bucharest University of Economic Studies, 010374 Bucharest, Romania; catalina.radu@man.ase.ro (C.R.); stanmiruna20@stud.ase.ro (M.-G.S.); dragomireugen19@stud.ase.ro (E.-M.D.)

**Keywords:** work–family conflict, perceived social support, meaning in work, well-being, employee engagement, performance, parental leave, sustainable workplaces

## Abstract

This study aims to examine the associations of work–family conflict, perceived social support, and meaning in work with well-being, engagement, and performance among employees returning from parental leave. The study is grounded in the Job Demands–Resources model and uses cross-sectional survey data from 1199 Romanian employees returning from parental leave. Structural equation modeling (SEM) was employed to test a comprehensive model linking job demands and resources to individual and organizational outcomes. Results indicate that both work-to-family and family-to-work conflict are negatively associated with employee well-being, whereas perceived social support and meaning in work are positively associated with well-being. Moreover, perceived social support and meaning in work are also positively associated with engagement, whereas well-being is not significantly associated with engagement. Both engagement and well-being are positively associated with self-reported performance. These findings highlight the relevance of social and psychological resources for employee functioning during the transition back to work after parental leave. The study contributes to the literature by integrating work–family dynamics and several dimensions of positive functioning within a comprehensive model of post-parental-leave reintegration.

## 1. Introduction

The transition back to work following parental leave can be challenging for both employees and organizations (Franzoi et al., 2024), and there are significant implications for long-term sustainability in the workplace (Piwowar-Sulej, 2021). Sustainable organizations are not only concerned with performance outcomes, but also with maintaining employee well-being, engagement, and supportive work environments over time (Kramar, 2022). Moreover, sustainable workplaces do not refer only to environmental sustainability (Aust et al., 2020), but also to the organizational capacity to support employees during vulnerable periods and major life transitions (Guest, 2017). In this context, the Job Demands–Resources model highlights the importance of considering both job demands and resources in supporting sustainable employee functioning (Xanthopoulou et al., 2007).

Parental leave should not be understood merely as a temporary interruption of professional activity, but rather as a transitional stage within the employment relationship (Godager & Riforgiate, 2025), during which the employee’s daily participation in organizational life is suspended, while the employee remains connected to the employer in legal, psychological, and professional terms (Ladge & Greenberg, 2015; OECD, 2023). In this sense, parental leave represents a period in which the formal work relationship is preserved (Ray et al., 2010), but the employee’s professional identity, organizational visibility, access to information, and sense of belonging may be affected by the distance from the workplace and by the new responsibilities associated with childcare (Buzzanell, 2024; Costantini et al., 2022; Cross et al., 2025).

In Romania, the main legal framework regulating parental leave is Government Emergency Ordinance No. 111/2010 on leave and the monthly allowance for child raising (Government of Romania, 2010), whose implementation is detailed in Government Decision No. 52/2011 (Government of Romania, 2011) and subsequently amended, including by Government Decision No. 865/2023 (Government of Romania, 2023). Some of the more recent legislative changes have also included Government Emergency Ordinance no. 164/2022 (Government of Romania, 2022) and have reflected the European principles concerning work–life balance for parents and carers, as reflected in Directive (EU) 2019/1158 (European Parliament and Council of the European Union, 2019). This legislative framework provides the institutional context within which professional reintegration after parental leave takes place in Romania.

In general, parental leave may be granted until the child reaches the age of two, or until the age of three in the case of a child with a disability (Government of Romania, 2010, 2011), which reflects the recognition that early childhood care requires an extended period of parental availability. An important feature of the Romanian framework is the non-transferable period reserved for the other parent, since, following recent legislative changes, the parent who did not initially request parental leave has an individual entitlement of at least two months, which cannot be transferred to the other parent if it is not used (Government of Romania, 2022, 2023). Beyond its administrative function, this provision has broader social relevance, as it seeks to encourage the involvement of both parents in early childcare and to reduce the unequal distribution of the professional costs associated with parenthood. From this perspective, parental leave functions not only as a social protection measure, but also as a public policy instrument aimed at supporting work–family balance, equal opportunities, and a more balanced distribution of caregiving responsibilities (Addati et al., 2022).

The effects of parental leave may be either positive or negative, depending largely on the quality of organizational support and on the way in which the return to work is prepared, communicated, and managed (Houston & Marks, 2003). When employees benefit from clear communication before their return, role clarification, access to updated information, managerial feedback, reasonable flexibility, realistic performance expectations, and a gradual reintegration plan, parental leave can contribute to strengthening loyalty, motivation, and professional engagement, because the employee does not experience the return as an isolated individual effort, but as a supported process of reconnection with the organization (Costantini et al., 2021; Houston & Marks, 2003).

In such circumstances, the return to work is less likely to be perceived as a rupture and more likely to become a process of progressive professional reconnection (Sumpter et al., 2024), in which employees are supported in regaining their rhythm, confidence, and effectiveness (Kokubo et al., 2023) without feeling immediate pressure to demonstrate full availability or competence after return (Correll et al., 2007). Conversely, when the return is treated as a simple administrative formality, without communication, role clarification, flexibility, or managerial attention, employees may experience poorer well-being and greater difficulties in resuming effective work, particularly when they are expected to return as though no significant personal, family, or professional transition had occurred (Okorn et al., 2025).

Employees may feel pressured to perform immediately at the same level as before the leave, may encounter difficulties in recovering information lost during their absence, may perceive a decline in professional visibility, or may feel that their family responsibilities are incompatible with organizational expectations (Torres et al., 2024). These difficulties can intensify the tension between the employee role and the parental role, generating work–family conflict or family–work conflict (Carlson et al., 2000; Mesmer-Magnus & Viswesvaran, 2005; Netemeyer et al., 1996). This issue becomes particularly relevant in the first months after return (Falletta et al., 2020), when employees’ time, energy, and emotional resources are simultaneously required by professional tasks and caregiving responsibilities (McCardel et al., 2022), and when even routine work demands may be experienced as more difficult because they overlap with sleep disruption (Insana et al., 2013), childcare logistics, emotional adjustment, and the need to rebuild professional confidence (Jaeckel et al., 2012).

At the same time, parental leave should not be interpreted exclusively as a career interruption or as a source of occupational vulnerability, because the experience of parenthood may also contribute to the development of transferable personal resources that can become valuable in professional contexts (Greenhaus & Powell, 2006). Responsibilities associated with childcare may strengthen skills and capacities such as time management, emotional intelligence, and the ability to manage competing demands, and these resources can become valuable in professional contexts (Ma et al., 2022). From this perspective, post-leave work outcomes may depend not only on the leave itself, but also on how the return to work is supported and managed by the organization (Gupta et al., 2025).

For organizations, parental leave has direct implications for human capital retention (Ladge et al., 2018), continuity of organizational knowledge (Bencsik et al., 2015), and trust in management practices, because the way in which the return is managed communicates to employees whether the organization values them only as productive workers or also recognizes their broader life responsibilities (Kossek et al., 2010).

A well-supported reintegration process can reduce the risk of absenteeism and staff turnover, whereas poor reintegration may lead to the loss of valuable employees, deterioration of the employment relationship, and lower organizational commitment (Medina-Garrido et al., 2020; Paustian-Underdahl et al., 2024; Wiese & Stertz, 2023). For this reason, parental leave should be integrated into human resource strategy as a process that begins before the employee’s absence, continues during the leave through appropriate communication, and becomes particularly important in the first months after return, when employees need support, clarity, flexibility, and recognition of their new family reality (Stumbitz et al., 2018).

Overall, the Romanian legal framework provides financial and legal protection for parents, but the actual effects of parental leave on well-being, engagement, and performance depend on the organizational practices that accompany the return to work. When supported through appropriate organizational practices, return from parental leave can contribute to human capital retention and sustainable employee functioning (Ladge et al., 2018; Stumbitz et al., 2018). It can therefore be approached as a natural stage in the professional path rather than as a deviation from the career trajectory (Kaushiva & Joshi, 2020). Parental leave can protect human capital and support sustainable performance when it is treated as a natural stage in the professional path, rather than as a deviation from the career trajectory. For this reason, the present study approaches the return after parental leave as a managerial, psychological, and social process and examines the associations among work–family conflict, perceived social support, meaning in work, well-being, engagement, and self-reported performance.

Perceived social support may act as a protective resource during this readjustment process (Feldman et al., 2004). Employees who perceive stronger support within their broader social environment may be better able to cope with stress and preserve psychological balance after returning from parental leave (Viswesvaran et al., 1999). From a sustainability perspective, social support is relevant to healthier work environments and to employees’ longer-term functioning rather than only to short-term productivity (Jolly et al., 2021; Nahum-Shani et al., 2011; Poon & Law, 2022).

Meaning in work is another relevant factor to consider in understanding employees’ adjustment after parental leave (Le Sueur & Boulton, 2021; Lysova et al., 2019). When employees perceive their work as purposeful and significant, they may be more motivated to reinvest energy in their professional roles (May et al., 2004). Meaningful work is not limited to task completion, but includes the perception that work contributes to personal growth, social value, or a greater good (Both-Nwabuwe et al., 2017; Martela & Pessi, 2018; Steger et al., 2012). This is important for sustainability because employees who experience meaning in work may be more likely to maintain engagement, well-being, and commitment over time (Lysova et al., 2019), even when facing the demands of balancing work and family responsibilities.

Employee well-being represents a central outcome in this framework, as it reflects employees’ psychological, emotional, and work-related functioning (Grant et al., 2007). Previous research has also highlighted that well-being should not be viewed merely as an outcome of favorable workplace conditions, as it is also associated with adaptability, performance, and long-term functioning in organizations (Boudrias et al., 2021; Radu & Stan, 2025). After parental leave, well-being may depend on the degree to which employees can balance role demands, access support, and experience their work as meaningful (Le Sueur & Boulton, 2021; Rapisarda et al., 2024). A multidimensional view of well-being is useful here because it includes not only job-related states, but also life well-being and psychological well-being (Page & Vella-Brodrick, 2009). This broader approach fits a sustainability-oriented perspective, as sustainable organizations need to protect employees’ health, emotional stability, and long-term capacity to function effectively at work (Tortia et al., 2022; Zheng et al., 2015).

Engagement also plays an important role in understanding employees’ return to work after parental leave (Costantini et al., 2021; Johnson et al., 2025). In the broader work-engagement literature, work engagement refers to a positive work-related state reflecting employees’ energy and involvement in their work (Schaufeli et al., 2002). Employees who feel supported and who perceive their work as meaningful may be more likely to experience energy, enthusiasm, and involvement in their work activities (Rich et al., 2010; Salanova et al., 2005). In this sense, engagement can be viewed as an important dimension of sustainable employee functioning, with relevance for both employees and organizations (Lu et al., 2023; Saks, 2006; Schaufeli et al., 2006). However, previous research also suggests that engagement may become difficult to sustain when employees experience elevated levels of stress, overload, or burnout symptoms, which can gradually undermine well-being and work functioning (Crawford et al., 2010; Olteanu & Radu, 2025; Radu et al., 2020).

Performance is generally understood in organizational research as employees’ effective contribution to work and organizational goals (Campbell & Wiernik, 2015). In the context of returning from parental leave, performance is relevant not only in terms of immediate productivity, but also in relation to sustainable employee functioning over time (La et al., 2026). In the present study, performance is examined through self-reported accomplishment, reflecting employees’ perceptions of success and effectiveness in their work. Accordingly, this operationalization captures a subjective aspect of performance rather than the full multidimensional construct of individual work performance described in the broader literature (Koopmans et al., 2011, 2014; Ramos-Villagrasa et al., 2019).

Previous research has examined work–family conflict, social support, meaning in work, well-being, engagement and performance in relation to work and family experiences, but these dimensions have often been addressed separately or in broader organizational contexts (Franzoi et al., 2024; Johnson et al., 2025).

The research gap addressed by the present study is the limited integration of these dimensions into a model applied to the return to work after parental leave, a stage in which employees may experience different combinations of work–family pressure, social and psychological resources, well-being, engagement and self-reported performance. Thus, the study approaches reintegration as a managerial, psychological and social process relevant to the sustainability of work.

The aim of the study is to examine the associations of work–family conflict, perceived social support and the meaning in work with well-being, engagement and performance of employees who return to work after parental leave. In this regard, the research examines the relationships between pressures across work and family roles and well-being, the associations of social and psychological resources with post-return adjustment, and the relationships of well-being and engagement with self-reported performance.

This study contributes to the literature on human resource management, work–family balance, and organizational sustainability by integrating work–family conflict, perceived social support, meaning in work, well-being, engagement, and self-reported performance into an integrated model of employees’ reintegration following parental leave.

## 2. Literature Review and Hypotheses Development

Professional reintegration after parental leave can be understood through the relationship between the demands that consume the employee’s resources and the resources that help them adapt, maintain their well-being, and function effectively at work. The Job Demands–Resource (JD-R) model provides a suitable framework for this analysis by distinguishing between two complementary processes: a health-impairment process, through which demanding work conditions may undermine employee well-being, and a motivational process, through which resources are associated with greater engagement and favorable work outcomes (Bakker et al., 2023; Schaufeli & Bakker, 2004). In the context of returning from parental leave, work–family conflict can be understood as a demand, whereas perceived social support and meaning in work represent resources that may be relevant to post-return adjustment (Crawford et al., 2010; Xanthopoulou et al., 2007). This relationship is particularly important because employees do not return to a neutral context, but to one in which professional and family roles continue to overlap and influence one another. Based on this theoretical framework, the present study examines the relationships between work–family conflict, perceived social support, meaning in work, well-being, engagement, and self-reported performance through the following hypotheses:

**H1a.**  *Work-to-family conflict is negatively associated with employee well-being.*

One of the most prominent challenges during this transition is the conflict between work and family, which reflects the incompatibility between professional and personal roles. Two directions of conflict are typically distinguished: work-to-family conflict, where work interferes with family life, and family-to-work conflict, where family responsibilities hinder work functioning (Carlson et al., 2000; Netemeyer et al., 1996). Both forms of conflict have been associated with lower well-being and greater strain (Allen et al., 2000; Kinnunen et al., 2003). Employees returning from parental leave may be particularly exposed to these tensions because professional demands coexist with continuing caregiving responsibilities (Falletta et al., 2020; McCardel et al., 2022). Work-to-family conflict occurs when work demands restrict employees’ ability to meet their family responsibilities. This form of conflict can be understood as a source of inter-role strain, in which work consumes time, energy, and psychological resources that would otherwise support personal and family life. Work interference with family is linked to adverse outcomes, including higher stress, health-related difficulties, and lower satisfaction, which may undermine employees’ overall well-being (Allen et al., 2000). The adverse effect of work-to-family conflict on psychological well-being may also be linked to insufficient recovery, because ongoing work-related interference can prevent employees from mentally disconnecting, relaxing, developing personally, and maintaining control over their free time, which may weaken the renewal of essential psychological resources (Sousa & Silva, 2025).

This relationship has also been supported through longitudinal evidence. Work–family conflict can predict lower employee well-being over time, based on both self-reports and peer evaluations. This suggests that the effects of conflict are not limited to subjective perceptions, but may also be reflected in employees’ observable functioning at work (Grant-Vallone & Donaldson, 2001). Further evidence supports this association in different occupational contexts. In a study conducted on teachers, work–family conflict was negatively related to occupational well-being, while psychological capital helped explain this relationship. Similar findings were reported in a sample of physicians, where work–family conflict was negatively associated with employee well-being through lower job satisfaction and reduced work engagement (Yang et al., 2024; Zhou et al., 2021).

More recent research has extended these findings across a variety of contemporary work contexts. During the COVID-19 period, work–family conflict was shaped by gender and parenthood, which highlights its relevance for employees with caregiving responsibilities (Reimann et al., 2022b). In addition, working from home has been shown to influence work–family conflict and employees’ functioning at the beginning of the following workday, suggesting that the spillover of work demands into family life may affect recovery and subsequent work performance (Darouei & Pluut, 2021). The family-side antecedents and consequences of work–family conflict further indicate that work-related interference extends beyond the occupational sphere, affecting family adjustment and individual outcomes (Reimann et al., 2022a). Occupational-health evidence links work–family conflict with depression among healthcare workers, while systematic review and meta-analytic evidence shows that work–family conflict is associated with health-related behaviors and outcomes (Peng et al., 2026; Zhang et al., 2023).

When work interferes with family life, employees may experience higher stress, exhaustion, and a decline in the psychological resources needed to maintain well-being (Allen et al., 2000; Grant-Vallone & Donaldson, 2001; Yang et al., 2024; Zhou et al., 2021). Taken together, these theoretical and empirical contributions indicate that work-to-family conflict is negatively associated with employee well-being.

Although the present hypothesis focuses on work-to-family conflict, the work–family interface is increasingly conceptualized as a bidirectional process in which pressures originating in either domain may interfere with functioning in the other. This broader perspective provides the rationale for examining family-to-work conflict separately in the following hypothesis.

**H1b.**  *Family-to-work conflict is negatively associated with employee well-being.*

Family-to-work conflict occurs when family-related responsibilities and demands negatively affect employees’ ability to adequately fulfill their professional roles. Difficulties originating in the family sphere may impair occupational functioning and contribute to unfavorable psychological outcomes (Frone et al., 1992). Recent evidence indicates that the effects of family-to-work conflict are not limited to employees’ mental state, but may also influence how effectively they perform their work tasks. Moreira et al. (2023) showed that interference from family into work was associated with lower task performance, while employee well-being reduced the strength of this negative relationship. Thus, well-being may represent an important personal resource that supports employees’ ability to remain effective at work when family demands interfere with professional responsibilities.

One explanation for this relationship is associated with the gradual depletion of employees’ personal resources. When individuals invest considerable emotional and cognitive effort in managing family responsibilities, fewer resources remain available for coping with work-related demands. Family interference with work has been linked to lower levels of well-being, suggesting that this type of conflict may increase stress and reduce employees’ psychological adjustment (Kinnunen et al., 2003). Support for this association has also been identified in the more recent literature across different occupational contexts. Family–work conflict was significantly associated with stress during the COVID-19 period, indicating that pressures stemming from the family domain may negatively affect employees’ psychological functioning. Similar findings were reported among nurses, where family–work conflict was negatively related to indicators of well-being, including life satisfaction and happiness. These findings suggest that tensions generated within the family environment may extend into the workplace and negatively influence employees’ overall well-being (El Keshky & Sarour, 2024; Elahi et al., 2022).

The existing body of theoretical and empirical literature therefore supports the assumption that family-to-work conflict is negatively associated with employee well-being. Employees exposed to persistent family-related pressures may experience elevated stress, emotional exhaustion, and difficulties in maintaining psychological balance when family demands interfere with occupational responsibilities (El Keshky & Sarour, 2024; Elahi et al., 2022; Frone et al., 1992; Kinnunen et al., 2003).

Studies that distinguish family-to-work conflict from the broader work–family conflict construct show that both work–family and family–work interference are associated with stress, confirming that pressures originating in the family domain may impair employees’ psychological functioning at work (Elahi et al., 2022). Both directions of conflict have also been associated with symptoms of anxiety and depression, with these relationships varying across social class positions (Ragnarsdóttir et al., 2026). Family-to-work conflict has been linked to work stress and innovative work behaviour among university teachers, indicating that family-based interference may affect both strain and work-related functioning (Bao et al., 2025). Family and work resources may buffer the mental-health implications of conflict across domains, while evidence from dual-earner parents shows that family-to-work conflict is associated with psychological distress and job satisfaction (Reimann & Diewald, 2022; Schnettler et al., 2024). This relationship may be particularly relevant after parental leave, when family responsibilities remain substantial while employees are re-establishing their professional routines.

**H2.**  *Perceived social support is positively associated with employee well-being.*

Perceived social support refers to the extent to which individuals feel supported and socially connected within their broader social environment. In the present study, the construct captures this broader interpersonal support rather than support exclusively from the organization, supervisors, or colleagues. As a psychosocial resource, perceived social support may help individuals cope with stress and maintain psychological balance during demanding transitions such as the return from parental leave. Social support can buffer the negative effects of stressors, facilitate coping, and enhance psychological well-being. In organizational contexts, supportive relationships contribute to healthier work environments and promote long-term sustainability.

Social support has frequently been identified as an important resource for maintaining employees’ psychological well-being. Employees who perceive that support is available from supervisors, colleagues, or the organization are generally better equipped to cope with occupational demands and stressful situations. Supportive workplace relationships may contribute to higher well-being by reducing psychological strain and strengthening employees’ sense of interpersonal balance within the work environment. Perceived support is therefore associated with more positive psychological outcomes and improved adjustment at work (Nahum-Shani et al., 2011). Recent findings show that support from the work environment can act together with employees’ personal resources, as perceived supervisor support may strengthen the positive relationship between work self-efficacy and psychological well-being, suggesting that employees who feel supported by their supervisors are better able to use their own confidence and abilities to cope with uncertainty and work-related pressure (Pires, 2025).

The beneficial role of perceived social support has also been highlighted in studies examining workplace resources and occupational health. Supportive organizational resources are positively associated with employee well-being and work-related functioning. In this context, social support may act as a protective mechanism that helps employees manage work demands more effectively while reducing the negative effects of stress. As a result, employees who perceive stronger support within the workplace are more likely to experience lower emotional strain and a more favorable psychological state (Nielsen et al., 2017).

More recent contributions provide additional support for this relationship across different professional settings. Workplace social support has been associated with positive employee outcomes, including improved affective experiences and healthier interpersonal relationships. Social support has also been positively related to job satisfaction and negatively associated with emotional exhaustion. These findings suggest that perceived social support may enhance employee well-being by strengthening psychological resources and reducing vulnerability to stress and burnout (Garmendia et al., 2023; Jolly et al., 2021). Perceived organizational support reflects employees’ perception that the organization values their contributions and cares about their well-being, positioning support as a central component of the employment relationship (Eisenberger et al., 2020). Employee health and well-being at work are shaped not only by stressors and strains, but also by job resources, interpersonal conditions, and organizational environments (Sonnentag et al., 2023). The employee-organization relationship has gained renewed importance in the post-pandemic context, particularly in relation to health and well-being (Shore et al., 2025). Social support at work is associated with beneficial affective, relational, and individual outcomes, while both work and non-work support are relevant to employee well-being, with perceived organizational support shaping this relationship (Jolly et al., 2021; Le et al., 2023).

**H3.**  *Meaning in work is positively associated with employee well-being.*

Meaning in work reflects the extent to which individuals perceive their work as purposeful and significant. For employees returning from parental leave, meaning may act as a motivational anchor that helps them reintegrate into their professional roles and maintain a sense of purpose despite competing demands.

Meaning in work is widely regarded as an important contributor to employee well-being because it allows individuals to experience their professional activity as purposeful, valuable, and connected to their broader sense of self. Self-Determination Theory perspectives on meaningful work emphasize the relevance of basic psychological needs such as autonomy, competence, and relatedness (Allan et al., 2016). In contrast, the absence of meaning at work may weaken well-being, as work alienation involves feelings of powerlessness, meaninglessness, and disconnection from one’s own activity and from broader organizational or social purposes (Arnold et al., 2007).

Meaningful work also extends beyond the formal boundaries of the organization, as it can shape how individuals understand their personal lives, identities, and relationships with others. Experiences of meaningful work are often emotionally significant and may lead employees to reflect on the purpose of their professional activity and on the broader value of their contribution. Such experiences can strengthen a sense of human connection, solidarity, and personal significance, which may support well-being by allowing individuals to perceive work as part of a wider life narrative (Bailey et al., 2017).

The positive relationship between meaning in work and well-being can also be explained through the alignment between work, personal values, and identity. When employees perceive their work as consistent with who they are and what they value, they are more likely to experience authenticity, purpose, and intrinsic motivation. Meaningful work has been associated with stronger psychological well-being, greater job satisfaction, and a higher tendency to view work as an important and valuable part of life. Employees who perceive their work as contributing to a broader purpose may therefore experience higher well-being and stronger connection to their work group (Rosso et al., 2010; Steger et al., 2012). The relationship between meaningful work and employee well-being can also be understood through employees’ experience of thriving at work, since work perceived as meaningful may encourage vitality and learning, thereby strengthening psychological well-being, particularly when the work environment is less hostile (Karadas et al., 2025).

Meaningful work is connected with well-being and health through a eudaimonic perspective, emphasizing the role of purpose, significance, and personal growth in work-related functioning (Soren & Ryff, 2023). The experience of meaningful work is also linked to psychological needs in the context of underemployment, suggesting that meaning helps explain how employment conditions relate to employee adjustment (Kim & Allan, 2020). Longitudinal evidence on decent and meaningful work indicates that meaningful employment has implications for psychological functioning over time (Allan et al., 2020). The longitudinal directional associations between meaningful work and mental well-being further indicate that work meaning is connected to later psychological well-being (Herr et al., 2023). Decent work and meaningful work are also presented as central constructs for understanding work as a source of dignity, psychological need satisfaction, and well-being (Blustein et al., 2023).

**H4.**  *Perceived social support is positively associated with employee engagement.*

Beyond its association with well-being, perceived social support is also expected to be positively associated with employee engagement. This relationship may be particularly relevant after parental leave, when employees are re-establishing involvement in their professional role while continuing to manage caregiving responsibilities (Costantini et al., 2021; Johnson et al., 2025).

Employee engagement tends to increase when employees perceive their work environment as supportive and responsive to their needs. From a social exchange perspective, support received from supervisors, colleagues, or the organization may encourage employees to reciprocate through greater energy, attention, and emotional involvement in their work. Perceived organizational support and perceived supervisor support have therefore been identified as important antecedents of employee engagement (Saks, 2006).

This relationship is also consistent with the Job Demands–Resources model, which suggests that job resources activate motivational processes and support work engagement. Social support can help employees cope with job demands, sustain motivation, and remain involved in their tasks. Supportive work conditions may therefore contribute to higher levels of work engagement (Hakanen et al., 2006; Schaufeli & Bakker, 2004). In addition to providing practical and emotional resources, social support may also encourage a deeper form of work engagement by helping employees remain more attentive, focused, and mentally present in their professional activities, while psychological safety can strengthen this process by creating a work environment in which employees feel secure enough to invest energy, attention, and commitment in their tasks (Wu et al., 2025). More recent studies further confirm the relevance of perceived support for employee engagement. Employees who perceive stronger organizational care and recognition are more likely to report higher work engagement. Organizational and supervisory support also remain important during crisis-related work contexts, such as the COVID-19 pandemic, indicating that perceived support can sustain engagement even under conditions of uncertainty and pressure (Imran et al., 2020; Lee & Shin, 2023).

The existing literature therefore supports the assumption that perceived social support is positively associated with employee engagement. When employees perceive that they receive support from the organization, supervisors, and colleagues, they are more likely to report higher levels of work engagement (Hakanen et al., 2006; Imran et al., 2020; Lee & Shin, 2023; Saks, 2006; Schaufeli & Bakker, 2004).

Job resources, including organizational and social support, activate motivational processes that foster work engagement within the Job Demands–Resources framework (Bakker et al., 2023). Meta-analytic evidence within the same framework shows that work engagement is systematically associated with job resources (Mazzetti et al., 2023). Perceived organizational and supervisory support during the COVID-19 crisis have been linked to employee engagement, partly through work–life balance policy (Lee & Shin, 2023). Perceived organizational support has also been associated with work engagement among employees with children with disabilities, making this relationship particularly relevant for employees with substantial family responsibilities (Stefanidis & Strogilos, 2021). Perceived organizational support is further connected to employee engagement and affective commitment, which in turn relate to organizational citizenship behavior (Alshaabani et al., 2021).

**H5.**  *Meaning in work is positively associated with employee engagement.*

For employees returning from parental leave, meaningful work may be particularly relevant as they reconnect with their professional role and reconsider the place of work within a changed personal and family context.

Meaningful work has been shown to exhibit strong associations with key occupational outcomes, including work engagement, organizational commitment, and job satisfaction. These relationships indicate that meaningful work is closely connected to motivational and attitudinal outcomes, including work engagement (Allan et al., 2019). During the deployment of United States soldiers on a peacekeeping mission in Bosnia, personality hardiness was associated with higher levels of engagement in mission-related activities. Soldiers who showed a stronger tendency to perceive their work as meaningful were more likely to identify with the peacekeeping role, regard their responsibilities as significant, and display greater personal involvement and commitment to the mission. Furthermore, this heightened engagement and sense of meaning during deployment were later linked to positive developmental outcomes, including increased personal growth and improved stress-management capabilities, several months after the mission had concluded (Britt et al., 2001).

Likewise, the study of Fairlie suggests that meaningful work makes a substantial and multifaceted contribution to employee engagement. Characteristics associated with meaningful work demonstrated stronger associations with employee engagement and with a broad range of occupational outcomes when compared to other work-related characteristics. Furthermore, meaningful work characteristics were identified as the strongest independent predictors of employee engagement, highlighting their central role in shaping employees’ motivational and psychological connection to their work (Fairlie, 2011).

Meaningful work constitutes a significant dimension of occupational experience and demonstrates strong associations with both work engagement and organizational commitment. The constructs of meaning, engagement, and commitment are widely regarded as essential determinants of effective organizational functioning, highlighting the motivational significance of work within individuals’ lives. Given that the workplace represents a primary context for personal development, occupies a substantial proportion of individuals’ daily lives, and shapes patterns of engagement and broader perceptions of life meaning, it serves as a critical environment through which positive psychological outcomes may be fostered (Geldenhuys et al., 2014). Individual engagement in work is influenced by the extent to which employees perceive work situations as meaningful and psychologically safe, as well as by the availability of personal resources that can be invested in role performance (Kahn, 1990). In particular, psychological meaningfulness constitutes a central determinant of engagement, as it reflects the perceived value and significance of work-related activities, thereby fostering greater willingness to invest oneself in one’s role. When individuals experience higher levels of meaningfulness, they are more likely to engage fully in their work tasks. Accordingly, engagement is contingent not only on the perceived meaningfulness embedded in work contexts, but also on the availability of attentional, cognitive, and emotional resources that enable sustained involvement in work roles. Another perspective highlights the role of leadership in creating meaningful work, showing that responsible leaders can strengthen employee engagement by helping employees view their tasks as more purposeful and valuable, which may lead them to invest more energy, attention, and commitment in their work roles (Dong & Zhong, 2022).

Employees who perceive their work as meaningful tend to exhibit higher levels of organizational commitment and a reduced propensity for turnover intentions. In addition, such perceptions are consistently associated with elevated levels of work engagement and enhanced productivity relative to employees who experience lower levels of meaningfulness in their work. Conversely, the absence or erosion of perceived work meaning has been linked to adverse attitudinal outcomes, including increased cynicism, which may undermine both engagement and sustained organizational involvement (Van Wingerden & Van Der Stoep, 2018).

Meaningful work is positively associated with employee engagement, particularly when considered alongside job resources (Albrecht et al., 2021). Work meaning mediates the relationship between job crafting and work engagement, indicating that employees become more engaged when their work is experienced as meaningful (Letona-Ibañez et al., 2021). Meaningfulness of work has also been identified as a mediator between job crafting and work engagement among tour leaders (Guo & Hou, 2022). Meaningful work is connected with affective commitment to change, with work engagement contributing to this relationship (Faisaluddin et al., 2024). Meaningful work also functions as a mediating mechanism between person-job fit and work engagement, reinforcing the role of meaning in employees’ motivational attachment to work (Merdiaty, 2024).

**H6.**  *Employee well-being is positively associated with engagement.*

Employee well-being is expected to be positively associated with engagement, as employees who experience higher levels of psychological well-being may have greater energy and psychological resources available for involvement in their work. In order to explain the link between well-being and engagement, a broader conceptualization of “full engagement,” which integrates attitudinal dimensions such as organizational commitment and citizenship behaviors alongside employee psychological well-being, is likely to yield more favorable outcomes for both individuals and organizations. An exclusive focus on commitment and citizenship behaviors may neglect employees’ psychological health, thereby increasing the risk of adverse well-being outcomes and calling into question the sustainability of high levels of engagement over time (Robertson & Cooper, 2010). Longitudinal evidence further supports the assumption that employee well-being may contribute to effective work functioning over time, indicating that specific dimensions of well-being, including emotional health, purpose in life, character strengths, and social connectedness, are associated with lower levels of work distraction and more favorable job-related outcomes at a subsequent point in time (Fung et al., 2024).

Previous research has also documented close associations between work engagement and employee well-being, further supporting the conceptual distinctiveness of these two dimensions (Shimazu & Schaufeli, 2009; Shuck & Reio, 2014; Sonnentag, 2003). Contemporary research conceptualizes work engagement as a positive affective–motivational state that reflects employees’ energy, dedication, and psychological connection to their work (Hakanen & Kaltiainen, 2023). Work engagement is also conceptualized as a core positive work-related state within the social psychology of work, closely connected to employees’ psychological experience of their work roles (Bakker, 2022). Psychological well-being and psychological empowerment are relevant to work engagement and sustainable employability, indicating that well-being-related resources contribute to sustained engagement (Rahi, 2022). This association may be particularly relevant after parental leave, when employees resume their professional roles while continuing to manage substantial caregiving and family responsibilities. In this context, higher psychological well-being may be associated with a greater capacity to remain involved and engaged in work.

**H7.**  *Employee engagement is positively associated with performance.*

Employee engagement is expected to be positively associated with performance, as engaged employees tend to invest greater effort, persistence, and focus in their work.

Work engagement has been consistently associated with employee performance. Engagement may develop through positive psychological states, such as authenticity and self-efficacy, which are strengthened when employees use their personal strengths at work. Higher engagement is, in turn, associated with better performance, as engaged employees are more likely to invest cognitive, emotional, and behavioral resources in their work activities (Van Wingerden & Van Der Stoep, 2018). Evidence from public higher education institutions also suggests that employee engagement may contribute to performance at the organizational level, as engaged employees are more likely to show involvement, commitment, and sustained effort that can support broader institutional effectiveness and improve organizational outcomes (Gede & Huluka, 2024). Work engagement has also been linked to several organizational outcomes, including employee performance, organizational citizenship behaviors, and lower withdrawal intentions. Among the attitudinal variables examined, work engagement appeared to be the strongest predictor of performance, suggesting that highly engaged employees are more likely to display effective, productive, and constructive work behaviors (Allan et al., 2019).

The engagement–performance relationship is also consistent with the Job Demands–Resources framework, in which work engagement represents a motivational state associated with favorable work outcomes. Engaged employees may be more likely to sustain effort, focus, and psychological involvement in their work activities, providing a theoretical basis for expecting a positive association with performance.

Meta-analytic evidence shows that work engagement is positively related to performance across different conceptualizations of performance (Christian et al., 2011; Neuber et al., 2022). A comparative meta-analysis across public, semipublic, and private sectors further shows that work engagement is associated with attitudinal, behavioral, and performance outcomes (Borst et al., 2020). The engagement–performance relationship is also supported by meta-analytic evidence focused specifically on job performance (Corbeanu & Iliescu, 2023). Firm-level evidence from an ICT-consulting context indicates that work engagement relates to task performance through innovative work behavior (Van Zyl et al., 2021). The relationship between work engagement and job performance may also depend on psychological capital as a moderating factor (Yao et al., 2022). For employees returning from parental leave, engagement may be particularly relevant to performance as they re-establish their professional routines while continuing to manage substantial family responsibilities. Maintaining energy, focus, and involvement in work may therefore be especially important during post-leave reintegration.

**H8.**  *Employee well-being is positively associated with performance.*

Psychological well-being represents a stronger predictor of employee performance than job satisfaction. Broader indicators of happiness and psychological functioning appear to capture dimensions of employee experience that are more directly connected to effective performance outcomes. Unlike job satisfaction, which mainly reflects evaluative attitudes toward one’s job, psychological well-being includes wider affective and cognitive states that may exert a stronger influence on productivity and effectiveness. For this reason, job satisfaction may be insufficient as a proxy for workplace happiness, while psychological well-being may therefore represent an important predictor of performance (Wright & Cropanzano, 2000). In the post-parental-leave context, this association may be particularly relevant because effective work functioning occurs alongside continuing caregiving and adjustment demands (Falletta et al., 2020; McCardel et al., 2022).

The relationship between psychological health and job performance may be shaped by both dispositional and situational factors. Individual characteristics such as neuroticism and self-efficacy, together with role-related stressors, can influence employees’ psychological well-being and their capacity to perform effectively at work.

Employee well-being is positively and consistently associated with organizational performance across several dimensions. Employees with higher levels of well-being tend to show greater motivation, stronger organizational commitment, and increased engagement in work activities, which can contribute to both individual and collective performance. Higher well-being is also linked to improved productivity, more effective collaboration, and stronger customer-oriented behaviors. At the organizational level, higher employee well-being is associated with lower absenteeism and turnover, as psychologically healthy employees are more likely to remain committed to organizational goals (Krekel et al., 2019).

Higher levels of happiness and psychological well-being are associated with superior productivity. A positive relationship between emotional well-being and work performance has been consistently identified across several complementary experimental approaches, suggesting that happier individuals tend to be intrinsically more productive. Some experiments examined naturally occurring life events that generated significant variations in well-being, thereby allowing the analysis of large-scale emotional changes on productivity in real-life contexts. Other experiments relied on randomly induced positive emotional states within controlled laboratory settings, making it possible to isolate the causal impact of happiness on performance outcomes. Although each methodological approach possessed distinct limitations, the convergence of findings across multiple experimental designs reinforced the robustness of the relationship between well-being and productivity. Psychological well-being may enhance performance by improving concentration, motivation, and cognitive functioning, whereas unhappiness may act as a psychological distraction that reduces individuals’ capacity to effectively engage with work tasks. These findings underscore the importance of emotional well-being as a significant determinant of employee productivity and organizational effectiveness. Moreover, workplaces that foster employee well-being may benefit from increased productivity and more sustainable performance outcomes (Oswald et al., 2015).

The happy–productive worker thesis has been revisited from a eudaimonic perspective, showing that employee well-being and performance are meaningfully connected in organizational research (Peiró et al., 2021). Cross-cultural meta-analytic evidence further indicates that subjective well-being is associated with job performance (Salgado & Moscoso, 2022). Employee psychological well-being is also related to job performance through mediating and moderating mechanisms that help explain this relationship (Kundi et al., 2021). Evidence from software professionals during the COVID-19 pandemic shows that well-being and productivity are interrelated in contemporary work settings (Russo et al., 2021). Research synthesis on happiness and productivity further indicates that positive well-being is connected with productive work outcomes (Fang et al., 2026).

Overall, the literature supports the idea that returning to work after parental leave should be considered as an adaptation process in which pressures between roles are associated with lower well-being, while social support and meaning in work are associated with engagement and favorable work outcomes. This perspective goes beyond the strictly administrative approach of the return and positions reintegration as a strategic responsibility of human resources management, with implications for both the employee and the sustainability of the organization.

## 3. Materials and Methods

### 3.1. Participants and Procedure

This study employed a cross-sectional quantitative survey design to examine the relationships between work–family conflict, perceived social support, meaning in work, well-being, engagement, and performance among employees who had returned to work after parental leave.

Data were collected through an online questionnaire administered via Google Forms between October and December 2025. A non-probability sampling strategy combining convenience and snowball sampling was employed. The survey link was disseminated through professional networks, organizational contacts, and online communities relevant to parenting and employment, while participants were encouraged to share the questionnaire with other eligible individuals within their professional and personal networks. This approach was adopted because no centralized sampling frame exists for employees who have returned to work following parental leave in Romania, making probability-based recruitment difficult in this context. Because participation was based on an open survey link and voluntary dissemination across these channels, respondents self-selected into the study. Eligibility was assessed through a screening question at the beginning of the questionnaire asking participants to confirm that they had already returned to work after parental leave. Only eligible respondents proceeded to complete the survey.

Eligibility for parental leave in Romania is governed by Government Emergency Ordinance No. 111/2010 (Government of Romania, 2010). Under this framework, parental leave may be accessed not only by biological parents, but also under specific legal conditions, by other entitled persons, including in cases of adoption, guardianship, or placement. One of the main eligibility requirements is that applicants must have obtained taxable income for at least 12 months during the two years preceding the child’s birth or, where applicable, the event giving rise to the entitlement.

In order to ensure compliance with this inclusion criterion, only questionnaires from persons who indicated the existence of a current work regime after leave and who filled in the item referring to the number of months elapsed since their return were included in our sample. Therefore, the wording “returned to work after parental leave” does not refer to persons who are still on leave or to persons who intended to return, but to persons who had actually resumed their professional activity at the time of participating in the study.

In order to clarify the time frame covered by the phrase “return to work”, the questionnaire included a specific question on the number of months since the resumption of work. The sample was not limited only to the first months after the return, as the objective of the research was to capture the experience of reintegration in several stages of the post-leave period. However, this methodological option requires careful interpretation, as work–family conflict, perceived support, well-being, engagement, and performance can vary as the employee moves away from the time of return. For this reason, the time elapsed since return is explicitly reported in the characteristics of the sample, and the results should be understood as reflecting post-return experiences at different stages of professional adaptation.

Participation was voluntary, and respondents were informed about the purpose of the research, the anonymity of the responses and the use of data exclusively for academic purposes. No personal identification data was collected and the responses were analysed only in aggregate form.

As the questionnaire was distributed via open link, professional networks and online communities, it is not possible to accurately determine the total number of people who received, viewed or accessed the invitation to participate. Consequently, a conventional response rate cannot be calculated, and instead, the study transparently reports the final valid sample of 1199 respondents.

Respondents who cumulatively met the following criteria were included in the study: (1) they had experience of parental leave/parental leave; (2) have returned to work after this period; (3) were employed at the time of completing the questionnaire; (4) have completed the voluntary questionnaire; (5) provided sufficient responses for inclusion in the final database. The questionnaire included filter questions on returning to work after parental leave and current employment status. If the respondent had not returned to work after parental leave or was not employed, the completion of the questionnaire stopped. Incomplete responses, responses that did not correspond to the target population and cases in which the experience of returning to work after parental leave was not confirmed were excluded.

The majority of participants were employed full-time (91.6%), and worked primarily on-site (52.9%). Most participants were employed in the private sector (76.6%). The sample was predominantly married or in a partnership (96.8%). Participants’ mean age was 30.78 years (SD = 8.94), and the average number of children was 2.33 (SD = 1.84). More details regarding the sample characteristics are presented in Table 1.

Time since returning to work following parental leave was measured categorically. Most participants had returned to work more than 12 months before completing the survey (54.0%), while 20.5% had returned within the previous 0–3 months, 12.3% within 4–6 months, and 13.2% within 7–12 months. Consequently, the sample reflects employees at different stages of post-parental-leave work reintegration rather than only those in the immediate return-to-work period. To assess whether the main structural findings were sensitive to this temporal heterogeneity, a subgroup sensitivity analysis based on time since return is reported in Section 4.4.

### 3.2. Measures

All constructs were measured using multi-item scales assessed on five-point Likert-type scales:

- Work–family conflict and family–work conflict were measured using five items each. These scales capture the extent to which work interferes with family life and vice versa (Gutek et al., 1991; van Vianen, 1999);
- Perceived social support was assessed with three items adapted from the Relationships dimension of the PERMA Profiler, reflecting the extent to which participants perceived support from colleagues and their social environment (Butler & Kern, 2016);
- Meaning in work was measured using three items adapted from the Meaning dimension of the PERMA Profiler, capturing the perceived purpose and significance of one’s work (Butler & Kern, 2016);
- Well-being was assessed with three items adapted from the Positive Emotions dimension of the PERMA Profiler, reflecting positive emotional states and self-regard (Butler & Kern, 2016);
- Engagement was measured using three items adapted from the Engagement dimension of the PERMA Profiler, capturing vigor, absorption, and enthusiasm at work (Butler & Kern, 2016);
- Performance was assessed using three items adapted from the Accomplishment dimension of the PERMA Profiler, reflecting perceived goal attainment, competence, and satisfaction with one’s achievements (Butler & Kern, 2016).

The use of PERMA-derived measures was intentional, as the study sought to capture several complementary dimensions of positive functioning within a common conceptual framework. The variables operationalized through the PERMA-Profiler were therefore based on distinct dimensions of the instrument—Relationships, Meaning, Positive Emotions, Engagement, and Accomplishment (Butler & Kern, 2016). These dimensions were treated as conceptually distinct but related facets rather than as fully independent constructs. Accordingly, associations among the PERMA-derived variables should be interpreted within a shared conceptual framework and not as evidence of causal relations among entirely independent psychological constructs.

The research tool was a structured questionnaire that included demographic and occupational questions, return-to-work experience questions, items measured on Likert scales, and open-ended questions.

The questionnaire collected information on age, level of education, marital status, number of children, age of the youngest child, type of return to work, work regime, sector of activity, field, hierarchical level, seniority, size of the organization, geographical location, childcare arrangements, support of partner or other persons and overtime.

In addition, the questionnaire included open-ended questions through which respondents could describe the difficulties encountered in returning to work, the types of support considered useful, advice for other people in the process of reintegration and the public policy measures considered relevant.

Given the single-source, self-report nature of the data, the potential for common method bias was considered both procedurally and statistically. Thus, procedurally, participation was voluntary and anonymous, and informed consent was obtained. Statistically, Harman’s single-factor test was conducted. An unrotated exploratory factor analysis including all observed indicators yielded a first factor accounting for 32.0% of the total variance, below the commonly used threshold of 50% (Podsakoff et al., 2003), suggesting that common method bias is unlikely to substantially affect the study’s findings.

### 3.3. Data Analysis

All analyses were conducted in R (version 4.4.3) using RStudio (version 2026.01.1). Data were analyzed using structural equation modeling (SEM) with the lavaan package in R. The model was estimated using the robust maximum likelihood estimator (MLR), which accounts for potential deviations from normality.

A two-step approach was employed. First, a confirmatory factor analysis (CFA) was conducted to assess the measurement model. Second, the structural model was tested to examine the hypothesized relationships among constructs.

Model fit was evaluated using multiple indices, including the Comparative Fit Index (CFI), Tucker–Lewis Index (TLI), Root Mean Square Error of Approximation (RMSEA), and Standardized Root Mean Square Residual (SRMR). Following conventional guidelines, values of CFI and TLI above 0.90 and RMSEA and SRMR below 0.08 were considered indicative of acceptable fit.

In addition, reliability and validity were assessed using Cronbach’s alpha, composite reliability (CR), and average variance extracted (AVE). To assess whether the structural results were sensitive to the time elapsed since return to work, the model was also re-estimated separately for respondents who had returned within the previous 12 months and those who had returned more than 12 months earlier. This analysis was treated as a sensitivity check rather than as a formal test of between-group differences.

## 4. Results

### 4.1. Descriptive Statistics and Correlations

Table 2 presents the descriptive statistics, reliability coefficients, and correlations among the study variables. Work–family conflict and family–work conflict were moderately correlated (r = 0.51, *p* < 0.001). Both forms of conflict were negatively associated with well-being, engagement, and performance. Social support and meaning were strongly and positively related to well-being and engagement, highlighting their role as key psychological resources. Performance was positively associated with engagement (r = 0.37, *p* < 0.001), well-being (r = 0.32, *p* < 0.001), and social support (r = 0.40, *p* < 0.001).

### 4.2. Measurement Model

A confirmatory factor analysis (CFA) was conducted to evaluate the measurement model comprising seven latent constructs: work-to-family conflict, family-to-work conflict, well-being, engagement, social support, meaning, and performance. The measurement model demonstrated a good fit to the data, χ^2^(254) = 1340.04, *p* < 0.001, CFI = 0.933, TLI = 0.921, RMSEA = 0.054 (90% CI [0.051, 0.057]), and SRMR = 0.055.

All factor loadings were statistically significant (*p* < 0.001) and generally exceeded the recommended threshold of 0.60. Most standardized loadings were moderate to high, although one indicator of engagement showed a relatively low loading. These results are presented in Table 3.

Internal consistency was assessed using Cronbach’s alpha, with values ranging from 0.61 to 0.89 across constructs. All scales demonstrated acceptable to good reliability, including Work–family conflict (α = 0.88), Family–work conflict (α = 0.87), Social support (α = 0.77), Meaning (α = 0.88), Well-being (α = 0.89), and Performance (α = 0.75). The Engagement construct showed lower reliability and convergent validity than the remaining constructs (α = 0.61, CR = 0.62, AVE = 0.37). Although the AVE was below the recommended threshold, all standardized factor loadings were statistically significant. This pattern is consistent with previous validation studies of the PERMA-Profiler, in which engagement-related indicators, particularly those reflecting absorption, exhibited greater variability than the other PERMA dimensions (Butler & Kern, 2016). Given the theoretical relevance of engagement within both the PERMA and Job Demands–Resources frameworks, together with the satisfactory factor loadings and the overall stability of the measurement model, the original three-item specification was retained. One possible explanation is that engagement may be more variable during the transition back to work after parental leave, as employees simultaneously readjust to professional roles and new family responsibilities.

Convergent validity was supported for most constructs, with AVE values ranging from 0.37 (engagement) to 0.74 (well-being). Discriminant validity was assessed using both the Fornell–Larcker criterion and the heterotrait–monotrait ratio of correlations (HTMT)—Table 4 and Table 5. While the Fornell–Larcker criterion suggested substantial conceptual proximity among several theoretically related constructs, particularly engagement, social support, and well-being, all HTMT values remained below the conservative threshold of 0.85 (range = 0.145–0.803), supporting acceptable discriminant validity across the measurement model (Henseler et al., 2015).

### 4.3. Structural Model

**Figure 1:**
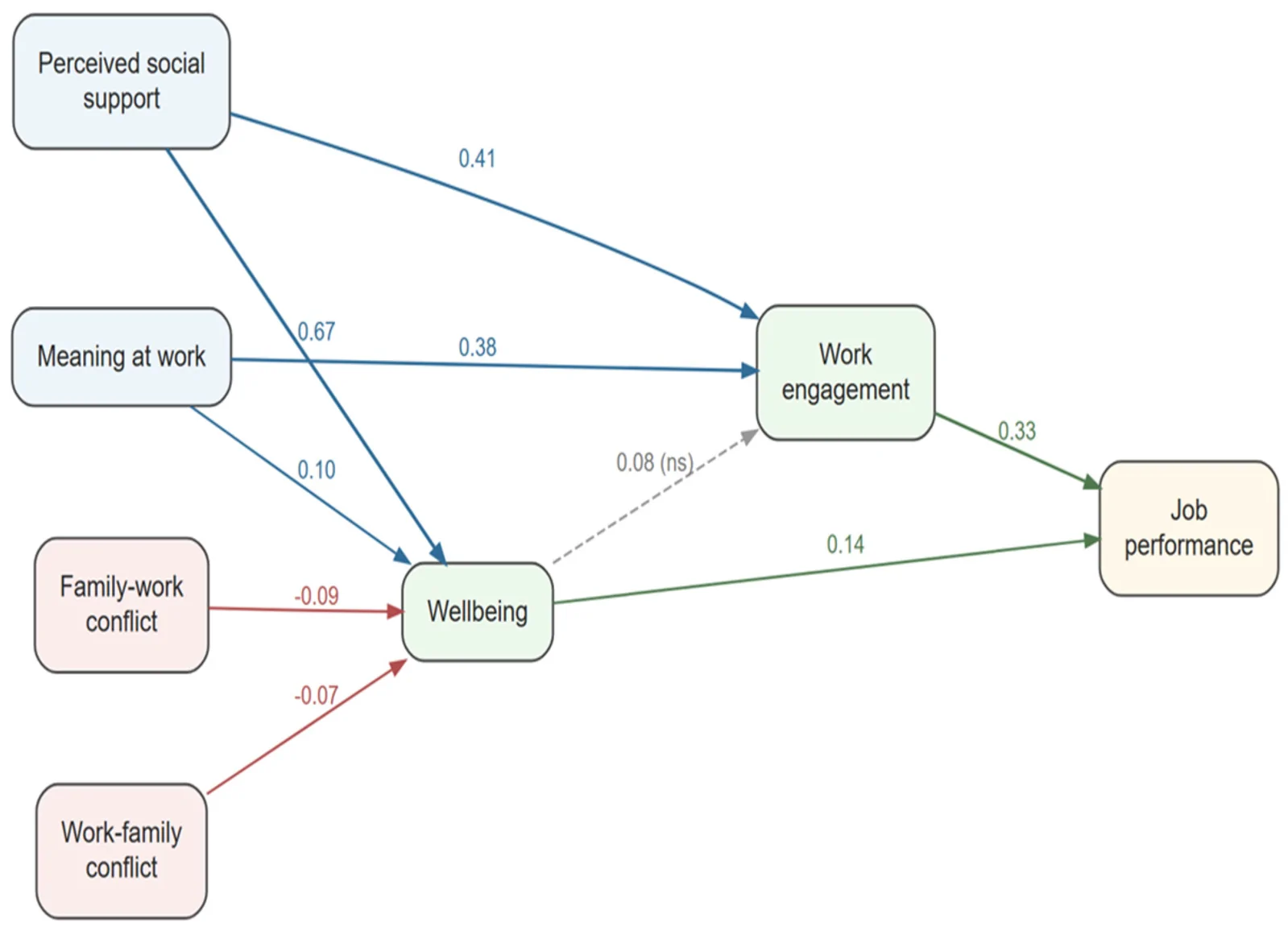
Structural equation modelling.

The structural model was tested using structural equation modeling (SEM) with robust maximum likelihood estimation. The structural model demonstrated an acceptable to good fit to the data, scaled χ^2^(260) = 1197.11, *p* < 0.001, robust CFI = 0.931, robust TLI = 0.920, robust RMSEA = 0.060, 90% CI [0.056, 0.063], and SRMR = 0.063. The model is presented in Figure 1.

Results indicated that both work-to-family conflict (β = −0.07, *p* = 0.032) and family-to-work conflict (β = −0.09, *p* = 0.009) were negatively associated with well-being.

Perceived social support was strongly and positively associated with well-being (β = 0.67, *p* < 0.001), while meaning showed a smaller positive association (β = 0.10, *p* = 0.002).

Contrary to expectations, well-being was not significantly associated with engagement (β = 0.08, *p* = 0.242). Engagement was positively associated with performance (β = 0.33, *p* < 0.001), while well-being showed a smaller positive association (β = 0.14, *p* = 0.024).

Engagement was a strong predictor of performance (β = 0.33, *p* < 0.001), while well-being also had a smaller but significant effect (β = 0.14, *p* = 0.024).

Overall, perceived social support and meaning showed substantial associations with well-being and engagement, while engagement showed the strongest association with self-reported performance among the modeled predictors. The model explained 65% of the variance in well-being, 59% in engagement, and 18% in performance. A synthesis of the model, by taking into account all the hypotheses, is presented in Table 6 (standardized coefficients are reported):

Taken together, the standardized coefficients indicate differences in the relative magnitude of the observed associations. Perceived social support showed the strongest association with well-being (β = 0.67), whereas the associations of work-to-family conflict (β = −0.07), family-to-work conflict (β = −0.09), and meaning (β = 0.10) with well-being were smaller in magnitude. Perceived social support (β = 0.41) and meaning (β = 0.38) showed associations of similar magnitude with engagement, while engagement showed a stronger association with self-reported performance (β = 0.33) than well-being did (β = 0.14). Within the proposed JD-R framework, this pattern is consistent with the relevance of social and psychological resources for positive employee functioning, although the cross-sectional design does not permit interpretation of these differences as causal effects.

The model explained approximately 65% of the variance in well-being, 59% in engagement, and 18% in performance.

### 4.4. Sensitivity Analysis by Time Since Return to Work

Given that 54.0% of respondents had returned to work more than 12 months before completing the survey, a sensitivity analysis was conducted to examine whether the main findings were dependent on the time elapsed since return. The structural model was re-estimated separately for respondents who had returned within the previous 12 months (n = 551) and those who had returned more than 12 months earlier (n = 648). The subgroup models were estimated using the same MLR estimator and model specification as the full-sample model. Table 7 compares the standardized structural coefficients across the full sample and the two subsamples:

All structural coefficients retained the same direction across the two subsamples. The principal associations involving social support, meaning, engagement, and performance were broadly consistent, although some smaller associations varied in magnitude and statistical significance. Notably, the non-significant association between well-being and engagement observed in the full sample was also non-significant in both subsamples. Engagement remained positively associated with performance in both groups. These findings indicate substantial consistency in the overall structural pattern, while also suggesting that the strength of some associations may vary depending on the time elapsed since return.

To complement the comparison of structural coefficients, model fit and explained variance were also examined across the full sample and the two time-since-return subsamples. These indicators are reported in Table 8.

Both subgroup models showed acceptable and closely comparable fit. Explained variance in performance was virtually identical across the two subsamples (R^2^ = 0.182 and 0.185), while the variance explained in well-being and engagement differed somewhat across groups. Overall, the sensitivity analysis suggests that the main pattern of findings was not attributable solely to respondents who were further removed from the return-to-work transition. However, given the cross-sectional design, differences between the two subsamples should not be interpreted as within-person changes over time.

## 5. Discussion

### 5.1. Interpretation of the Hypothesized Relationships (H1a–H8)

Drawing on a demands–resources perspective, the findings provide a coherent picture of how demands, reflected in work–family conflict, and psychological and social resources are associated with employee functioning in the context of returning to work after parental leave. Overall, psychological and social resources showed stronger associations with the outcomes examined than the two forms of work–family conflict.

The findings supported hypothesis H1a, showing that work-to-family conflict is negatively associated with employee well-being. This suggests that when professional responsibilities interfere with family life, employees returning from parental leave may experience lower psychological well-being. Such interference may be accompanied by greater stress, emotional strain, and a reduced capacity to maintain balance between work and family roles. This result is consistent with prior studies showing that work-to-family conflict can undermine employee well-being over time (Allen et al., 2000; Grant-Vallone & Donaldson, 2001; Yang et al., 2024; Zhou et al., 2021). Thus, work-related interference with family life may represent an important obstacle to employees’ sustainable adjustment after parental leave.

The results also provided support for hypothesis H1b, indicating that family-to-work conflict is negatively related to employee well-being. In other words, when family responsibilities interfere with professional functioning, employees may experience greater psychological strain and lower well-being. This finding supports the view that pressures originating in the family domain can extend into the workplace and reduce employees’ emotional and cognitive resources (Frone et al., 1992; Kinnunen et al., 2003). Similar evidence has been reported in more recent studies linking family-to-work conflict with stress, reduced life satisfaction, and lower happiness (El Keshky & Sarour, 2024; Elahi et al., 2022). Therefore, family-related demands may complicate the return-to-work process by weakening employees’ psychological adjustment.

Hypothesis H2 was confirmed, as perceived social support was positively associated with employee well-being. Employees who perceived higher levels of social support reported better psychological well-being after returning from parental leave. This finding highlights the protective role of supportive relationships, which may reduce stress, strengthen feelings of belonging, and help employees cope more effectively with occupational demands. Previous research similarly emphasizes that workplace social support is linked to more favorable psychological outcomes, higher job satisfaction, and lower emotional exhaustion (Garmendia et al., 2023; Jolly et al., 2021; Nahum-Shani et al., 2011; Nielsen et al., 2017). Accordingly, perceived social support appears to be a key resource for sustainable employee adjustment.

Hypothesis H3 was also confirmed, showing that meaning in work is positively associated with employee well-being. For employees returning from parental leave, perceiving work as purposeful and aligned with their values may facilitate reconnection with the professional role while supporting psychological well-being. The finding is consistent with Self-Determination Theory perspectives on meaningful work, which emphasize the relevance of basic psychological needs such as autonomy, competence, and relatedness (Allan et al., 2016), and other studies that showed that meaningful work is associated with psychological well-being and job satisfaction (Steger et al., 2012). In this context, meaning in work may also help employees integrate their professional role with their broader personal and family identity.

The findings further supported hypothesis H4, demonstrating a positive association between perceived social support and employee engagement. For employees returning from parental leave, perceived support may make it easier to invest energy and attention in the professional role while managing continuing family demands. This result is consistent with the social exchange perspective, which suggests that employees tend to respond to perceived organizational and supervisory support through stronger engagement (Saks, 2006). It also aligns with the broader Job Demands–Resources logic, according to which social resources can support motivation and work engagement (Hakanen et al., 2006; Schaufeli & Bakker, 2004). Thus, perceived social support may be particularly relevant during post-parental-leave reintegration, when employees are simultaneously rebuilding their professional involvement and managing continuing family responsibilities.

The results also show that hypothesis H5, which proposed a positive relationship between meaning in work and employee engagement, was supported. Employees who experience their work as meaningful appear more likely to invest energy, attention, and emotional involvement in their work roles. This finding is consistent with the argument that psychological meaningfulness is an important condition for engagement (Kahn, 1990) and aligns with previous studies linking meaningful work to engagement and organizational commitment (Allan et al., 2019; Fairlie, 2011; Geldenhuys et al., 2014).

Contrary to hypothesis H6, employee well-being was not significantly associated with engagement. This result is particularly interesting because it diverges from the common assumption that employees with higher well-being are naturally more engaged in their work (Robertson & Cooper, 2010). Although previous research has reported associations between engagement and well-being (Shimazu & Schaufeli, 2009; Shuck & Reio, 2014; Sonnentag, 2003), our findings do not indicate a significant association between well-being and engagement once perceived social support and meaning in work are taken into account. Within the proposed model, engagement was more strongly associated with perceived social support and meaning than with well-being. This suggests that feeling well and feeling engaged may represent related but distinct aspects of the post-return work experience, with well-being not necessarily corresponding to the energy, dedication, and involvement captured by engagement. This distinction may be particularly relevant after parental leave, when employees may experience relatively positive well-being while still facing competing work and family demands that may limit the energy and involvement available for their professional role.

Hypothesis H7 was supported, with employee engagement positively associated with self-reported performance. Higher engagement was associated with higher self-reported performance in the present sample. This pattern is consistent with previous research and meta-analytic evidence linking engagement with job performance (Christian et al., 2011; Corbeanu & Iliescu, 2023; Neuber et al., 2022).

The relationship between employee well-being and self-reported performance was also confirmed, providing support for hypothesis H8. This association may be particularly relevant after parental leave, when maintaining psychological well-being may coincide with a greater capacity to manage competing work and family demands and sustain effective functioning at work. The finding is consistent with previous research linking psychological well-being with performance (Oswald et al., 2015; Wright & Cropanzano, 2000).

Looking across the structural relationships, perceived social support showed the strongest association with well-being and was also positively associated with engagement, while meaning in work was positively associated with both well-being and engagement. Engagement, in turn, showed the strongest association with self-reported performance among the predictors included in the model, whereas well-being showed a smaller positive association with performance.

Taken together, the findings are better interpreted as a pattern of associations among related dimensions than as a demonstrated causal sequence. Within the proposed model, perceived social support and meaning showed stronger associations with engagement than well-being did, while engagement showed the strongest association with self-reported performance. Because several of these variables were operationalized using related dimensions of the PERMA-Profiler and the data are cross-sectional, these paths should not be interpreted as evidence that one dimension causally produces another.

### 5.2. Theoretical Implications

From a theoretical perspective, the results strengthen the interpretation of the return after parental leave as a process of professional, psychological and social transition, not just as an administrative resumption of activity. By integrating work–family conflict, perceived social support, meaning in work, well-being, engagement and self-reported performance into a single model, the study extends the demands–resources logic to a context in which professional demands coexist with substantial family responsibilities.

Within the proposed model, perceived social support and meaning showed stronger associations with well-being and engagement than the two forms of work–family conflict. From a Job Demands–Resources perspective, this pattern is consistent with the relevance of resources during the return-to-work transition, while the comparatively small coefficients for work–family conflict suggest that demands represent only one component of post-parental-leave adjustment. In addition, the results support a more nuanced understanding of the relationship between well-being, engagement and performance. This distinction may be particularly relevant after parental leave, when professional demands coexist with continuing caregiving responsibilities, making it theoretically important to consider both demands and the social and psychological resources available to employees.

The non-significant association between well-being and engagement also suggests that these dimensions capture related but distinct aspects of employee functioning. Within the proposed model, higher well-being was not necessarily accompanied by higher engagement once perceived social support and meaning were taken into account. At the same time, engagement was positively associated with self-reported performance. This pattern is broadly consistent with the JD-R distinction between health-impairment and motivational processes (Bakker et al., 2023; Schaufeli & Bakker, 2004). However, the cross-sectional design and the conceptual proximity of the PERMA-derived dimensions do not allow engagement to be interpreted as a causal mechanism linking resources to performance.

The model accounted for approximately 65% of the variance in well-being, 59% of the variance in engagement, and 18% of the variance in self-reported performance. These values indicate that the proposed model accounts for a substantial proportion of variance in well-being and engagement, whereas a larger proportion of variance in self-reported performance remains unexplained by the variables included in the model.

Our study contributes to the literature on sustainable workplaces by integrating work–family conflict and psychological and social resources into a unified model of employee functioning during the transition from parental leave to work. The findings identify perceived social support as the resource most strongly associated with well-being and show that meaning and engagement are also relevant within this network of relationships. At the same time, the use of related PERMA-derived dimensions highlights the value of examining positive functioning as a set of conceptually distinct but interconnected experiences rather than as a sequence of fully independent mechanisms.

### 5.3. Practical Implications

From a practical perspective, the results indicate that organizations should treat the return from parental leave as a planned process, not as a one-off administrative moment. Helpful interventions may include appropriate contact before return, a formal reintegration discussion, role clarification, task prioritization, workload recalibration in the first few months, and regular feedback. These measures do not imply lowering performance standards, but creating the conditions under which standards can be sustained without exhaustion.

At the managerial level, organizations can contribute to a supportive return-to-work environment through concrete behaviors such as empathic communication, availability for clarifications, reasonable flexibility, constructive feedback, and recognition of caregiving realities. Direct managers have an important role because formal HR policies become meaningful to employees through their consistent implementation in daily interactions (Bowen & Ostroff, 2004). Therefore, training managers in family-supportive behaviors (Hammer et al., 2011) and in practices that promote psychological safety (Edmondson, 1999) may represent a useful component of organizational reintegration practices.

Because the sample included employees at different stages after return, reintegration practices should avoid assuming that employees’ needs are uniform across the post-leave period. The heterogeneity of the sample in time since return and work arrangements also cautions against assuming that a single reintegration practice will be equally relevant to all returning employees. Role clarification, reasonable workload adjustment, supportive relationships, meaningful work, and opportunities for contribution may all be relevant, while longitudinal research is needed to determine whether their relative importance changes over time.

The non-significant association between well-being and engagement also suggests a practical consideration: reintegration programs may benefit from addressing well-being and work engagement as related but distinct aspects of employee functioning. Thus, well-being can be supported in parallel with active engagement, and performance can be considered as part of a broader professional reconnection process, rather than only as an immediate indicator of productivity.

In terms of practical implications, our findings suggest that interventions supporting employees returning from parental leave may benefit from combining efforts to reduce work–family conflict with practices that strengthen supportive relationships and opportunities to experience work as meaningful. Organizations can seek to reduce work–family conflict through flexible arrangements and supportive policies, while also creating conditions for supportive workplace relationships through leadership practices, team integration, and mentoring, and for meaningful work through job crafting, value alignment, and opportunities for contribution. A combined focus on reducing demands and strengthening resources may therefore provide a useful basis for supporting longer-term employee functioning and organizational sustainability.

### 5.4. Limitations and Future Research

We would like to acknowledge a series of limitations. First, the cross-sectional design limits causal inferences, and the hypothesized directions of the paths are theoretically grounded, but reciprocal or reverse relations cannot be ruled out. Longitudinal designs following employees from the final months of leave through the first year after the return would allow the temporal ordering of conflict, resources, well-being, engagement and performance to be tested directly.

Second, all constructs were measured through self-report from a single source, which raises the possibility of common method bias. We addressed this both procedurally, through voluntary and anonymous participation, and statistically, through Harman’s single-factor test. The first factor accounted for 32.0% of the total variance, indicating that no single factor dominated the covariance among the measures. Nevertheless, future studies would benefit from supervisor-rated performance or objective performance indicators.

Third, the non-probability sampling strategy, combining convenience and snowball sampling, and the impossibility of determining a conventional response rate limit the generalizability of our findings. The open-link recruitment process may also have introduced self-selection bias, as employees with particularly salient positive or negative return-to-work experiences may have been more motivated to participate. Because no sampling frame or information on non-respondents was available, the direction and magnitude of this potential bias cannot be determined. Organizational panels or registry-based sampling frames would provide more representative samples of employees returning from parental leave.

Fourth, another limitation concerns the heterogeneity in time since return to work. More than half of the respondents (54.0%) had returned to work more than 12 months before completing the survey, meaning that the sample captures different stages of post-parental-leave reintegration rather than exclusively the immediate return-to-work period. Although the sensitivity analysis showed that the principal pattern of structural associations was broadly consistent across respondents who had returned within the previous 12 months and those who had returned earlier, the cross-sectional design does not allow these differences to be interpreted as changes occurring over time. Future longitudinal research should follow employees from the initial return-to-work period through later stages of reintegration.

Fifth, gender was not collected as part of the demographic information in this study. This represents a limitation, as potential gender differences in post-parental-leave work reintegration could not be examined. Future research should collect gender information to allow a more precise characterization of the sample and, where sample sizes permit, examine potential gender-related differences in post-parental-leave work reintegration.

Sixth, although we did collect several demographic and occupational characteristics, such as education level, employment sector, work arrangement, organizational size, and tenure, these variables were not incorporated into the structural model, which was designed to focus on the core psychological relationships among work–family conflict, social support, meaning, well-being, engagement, and performance following parental leave. Future research could examine whether these contextual characteristics act as moderators or boundary conditions of the relationships identified in this study, for example through multi-group SEM analyses or the inclusion of control variables.

Seventh, five variables in the structural model—perceived social support, meaning, well-being, engagement, and performance—were operationalized using distinct dimensions derived from the PERMA-Profiler. Although these dimensions are conceptually distinguishable, they originate from the same broader instrument and are theoretically expected to be related. The observed associations among these variables may therefore partly reflect their conceptual and measurement proximity and should not be interpreted as relationships among fully independent constructs. This issue is particularly relevant for engagement, which showed weaker measurement properties, including modest reliability and convergent validity and one indicator with a relatively low loading. Accordingly, findings involving engagement should be interpreted with particular caution. Future studies should replicate the proposed relationships using dedicated construct-specific measures, particularly established work–engagement and work–performance scales.

Eighth, our study was conducted in a single country. Romania’s parental-leave framework, which allows leave of up to two years and in which parental leave is predominantly taken by mothers, may differ from other national contexts. Replication across different parental-leave regimes would help clarify which of the present findings are context-specific and which generalize across institutional settings.

It would also be valuable to further investigate why well-being was not significantly associated with engagement in the present model and whether this pattern holds across different occupational or cultural settings. In addition, examining potential moderators, such as organizational policies, job flexibility, organizational culture, or leadership style, may provide deeper insights into how sustainable work environments can be effectively cultivated.

## 6. Conclusions

Returning to work after parental leave is a transition in which the usual architecture of employee functioning is rearranged. In our sample of 1199 Romanian employees, perceived social support was strongly associated with well-being, meaning was positively associated with engagement, and engagement showed the strongest association with self-reported performance, while well-being was not significantly associated with engagement.

Our findings suggest that social and psychological resources are strongly associated with employee functioning during the return from parental leave. Within the proposed model, perceived social support showed particularly strong associations with well-being and engagement, while engagement was positively associated with self-reported performance. These relationships should be interpreted as associations rather than as evidence of a causal sequence, particularly given the cross-sectional design and the conceptual proximity of the PERMA-derived dimensions.

Overall, our findings suggest that sustainable workplace functioning is associated not only with lower work–family conflict but also with the availability of supportive relationships and meaningful work. Organizations supporting employees returning from parental leave may therefore benefit from paying attention to both work–family demands and the social and psychological resources associated with more favorable post-return experiences.

## Figures and Tables

**Table 1:** Sample characteristics.

Characteristic	Category	n	%
Education	High school	55	4.6
	Post-secondary	32	2.7
	Bachelor’s degree	403	33.6
	Master’s degree	672	56.0
	PhD	37	3.1
Marital status	Married/cohabiting	1161	96.8
	Single	13	1.1
	Divorced/widowed	25	2.1
Work schedule after return	Full-time	1098	91.6
	Part-time	53	4.4
	Flexible schedule	48	4.0
Current work arrangement	On-site	634	52.9
	Hybrid	428	35.7
	Remote	137	11.4
Sector	Private	918	76.6
	Public	267	22.3
	NGO	14	1.2
Time since returning to work after parental leave	0–3 months	246	20.5
	4–6 months	147	12.3
	7–12 months	158	13.2
	More than 12 months	648	54.0
	Total	1199	100.0

Source: Authors’ own research.

**Table 2:** Descriptive statistics, reliability, and correlations.

Variable	M	SD	α	CR	AVE	1	2	3	4	5	6	7
1. Work–family conflict	2.93	1.08	0.88	0.88	0.61	-						
2. Family work conflict	2.27	1.05	0.88	0.88	0.60	0.51 ***	-					
3. Engagement	3.63	0.83	0.61	0.62	0.37	−0.23 ***	−0.27 ***	-				
4. Well-being	3.78	0.99	0.89	0.89	0.74	−0.36 ***	−0.42 ***	0.60 ***	-			
5. Social support	3.83	0.92	0.77	0.77	0.53	−0.34 ***	−0.40 ***	0.68 ***	0.79 ***	-		
6. Meaning	3.71	1.11	0.88	0.87	0.70	−0.23 ***	−0.31 ***	0.65 ***	0.53 ***	0.57 ***	-	
7. Performance	4.54	0.59	0.75	0.76	0.52	−0.15 ***	−0.39 ***	0.37 ***	0.32 ***	0.40 ***	0.33 ***	-

Note. N = 1199. M = mean; SD = standard deviation; α = Cronbach’s alpha; CR = composite reliability; AVE = average variance extracted. *** *p* < 0.001. Source: Authors’ own research.

**Table 3:** Standardized factor loadings for the measurement model.

Construct	Item	Standardized Loading	*p*-Value
Work–family conflict	W-F1. My work prevents me spending sufficient quality time with my family.	0.791	<0.001
	W-F2. There is no time left at the end of the day to do the things I’d like at home (e.g., chores and leisure activities).	0.644	<0.001
	W-F3. My family misses out because of my work commitments.	0.883	<0.001
	W-F4. My work has a negative impact on my family life.	0.856	<0.001
	W-F5. Working often makes me irritable or short tempered at home.	0.69	<0.001
Family–work conflict	F-W1. My work performance suffers because of my personal and family commitments.	0.772	<0.001
	F-W2. Family related concerns or responsibilities often distract me at work.	0.781	<0.001
	F-W3. If I did not have a family, I would be a better employee.	0.715	<0.001
	F-W4. My family has a negative impact on my day to day work duties.	0.785	<0.001
	F-W5. It is difficult to concentrate at work because I am so exhausted by family responsibilities.	0.807	<0.001
Well-being (Positive emotions)	P1. In general, I feel happy.	0.869	<0.001
	P2. I have many positive experiences.	0.902	<0.001
	P3. I feel good about myself.	0.799	<0.001
Engagement	E1. I feel completely absorbed in what I do.	0.399	<0.001
	E2. Time passes quickly when I am working on something that is important to me.	0.549	<0.001
	E3. My professional activities make me feel energized.	0.807	<0.001
Social Support (Relationships)	R1. I have warm and satisfying relationships with others.	0.784	<0.001
	R2. There are people in my life who genuinely support me.	0.631	<0.001
	R3. I feel socially connected.	0.762	<0.001
Meaning	M1. I have a clear purpose in life.	0.587	<0.001
	M2. My work contributes to something meaningful.	0.927	<0.001
	M3. I feel that what I do is important.	0.951	<0.001
Performance (adapted from Accomplishment)	A1. I feel successful in achieving my goals.	0.848	<0.001
	A2. I feel competent in my professional activities	0.738	<0.001
	A3. I am satisfied with what I have accomplished so far.	0.647	<0.001

Note. All loadings were estimated using robust maximum likelihood (MLR) in the confirmatory factor analysis. The Engagement indicator E1 showed a loading below the conventional 0.50 threshold, yet it was kept in the analysis, as explained below. Source: Authors’ own research.

**Table 4:** Discriminant validity: Fornell–Larcker criterion.

Variable	1	2	3	4	5	6	7
1. Conflict (W-F)	**0.778**						
2. Conflict (F-W)	0.508	**0.773**					
3. Engagement	−0.229	−0.267	**0.608**				
4. Well-being	−0.362	−0.419	0.601	**0.858**			
5. Social support	−0.341	−0.400	0.675	0.788	**0.729**		
6. Meaning	−0.228	−0.307	0.644	0.524	0.569	**0.838**	
7. Performance	−0.269	−0.516	0.737	0.683	0.715	0.747	**0.720**

Note. Diagonal values represent the square root of AVE (in bold in the manuscript); off-diagonal values are inter-construct correlations. Source: Authors’ own research.

**Table 5:** Discriminant validity: heterotrait–monotrait ratio of correlations (HTMT).

Variable	1	2	3	4	5	6	7
1. Conflict (W-F)	–						
2. Conflict (F-W)	0.525	–					
3. Engagement	0.261	0.145	–				
4. Well-being	0.390	0.427	0.543	–			
5. Social support	0.361	0.398	0.541	0.803	–		
6. Meaning	0.275	0.346	0.655	0.640	0.665	–	
7. Performance	0.282	0.511	0.696	0.686	0.691	0.818	–

Note. All values are below the conservative threshold of 0.85, supporting discriminant validity (Henseler et al., 2015). Source: Authors’ own research.

**Table 6:** Structural Model Results (SEM): Standardized Path Coefficients.

Hypothesis	Path	β (Std.)	*p*-Value	Result
H1a	Work-to-family conflict → Well-being	−0.07	0.0032	Supported
H1b	Family-to-work conflict → Well-being	−0.09	0.009	Supported
H2	Social support → Well-being	0.67	<0.001	Supported
H3	Meaning → Well-being	0.10	0.002	Supported
H4	Social support → Engagement	0.41	<0.001	Supported
H5	Meaning → Engagement	0.38	<0.001	Supported
H6	Well-being → Engagement	0.08	0.242	Not supported
H7	Engagement → Performance	0.33	<0.001	Supported
H8	Well-being → Performance	0.14	0.024	Supported

Source: Authors’ own research.

**Table 7:** Sensitivity analysis of structural paths by time since return to work.

Structural Path	Full Sample β (*p*)	≤12 Months β (*p*)	>12 Months β (*p*)
Work-to-family conflict → Well-being	−0.066 (0.032)	−0.050 (0.327)	−0.082 (0.030)
Family-to-work conflict → Well-being	−0.090 (0.009)	−0.125 (0.017)	−0.051 (0.271)
Social support → Well-being	0.674 (<0.001)	0.614 (<0.001)	0.736 (<0.001)
Meaning → Well-being	0.100 (0.002)	0.124 (0.011)	0.081 (0.078)
Well-being → Engagement	0.081 (0.242)	0.077 (0.368)	0.135 (0.245)
Social support → Engagement	0.414 (<0.001)	0.488 (<0.001)	0.296 (<0.001)
Meaning → Engagement	0.382 (<0.001)	0.351 (<0.001)	0.414 (<0.001)
Engagement → Performance	0.332 (<0.001)	0.362 (<0.001)	0.319 (<0.001)
Well-being → Performance	0.135 (0.024)	0.093 (0.332)	0.156 (0.037)

Note. Standardized coefficients are reported. The subgroup analysis represents a sensitivity analysis; differences between coefficients were not formally tested and should not be interpreted as statistically significant between-group differences. Source: Authors’ own research.

**Table 8:** Model fit and explained variance in the sensitivity analysis.

Indicator	Full Sample	≤12 Months	>12 Months
N	1199	551	648
Scaled χ^2^ (df = 260)	1197.114	677.708	805.704
*p*	<0.001	<0.001	<0.001
Robust CFI	0.931	0.934	0.927
Robust TLI	0.920	0.924	0.916
Robust RMSEA	0.060	0.059	0.061
90% CI for RMSEA	[0.056, 0.063]	[0.053, 0.064]	[0.056, 0.066]
SRMR	0.063	0.065	0.066
R^2^ Well-being	0.646	0.600	0.699
R^2^ Engagement	0.589	0.653	0.535
R^2^ Performance	0.183	0.182	0.185

Note. Models were estimated using robust maximum likelihood (MLR). Robust fit indices are reported for CFI, TLI, and RMSEA. Source: Authors’ own research.

## Data Availability

The original contributions presented in this study are included in the article. Further inquiries can be directed to the corresponding author.
